# Novel markers for high-throughput protoplast-based analyses of phytohormone signaling

**DOI:** 10.1371/journal.pone.0234154

**Published:** 2020-06-04

**Authors:** Silke Lehmann, Ana Dominguez-Ferreras, Wei-Jie Huang, Katherine Denby, Vardis Ntoukakis, Patrick Schäfer

**Affiliations:** 1 School of Life Sciences, The University of Warwick, Coventry, England, United Kingdom; 2 Warwick Integrative Synthetic Biology Centre, The University of Warwick, Coventry, England, United Kingdom; National Taiwan University, TAIWAN

## Abstract

Phytohormones mediate most diverse processes in plants, ranging from organ development to immune responses. Receptor protein complexes perceive changes in intracellular phytohormone levels and trigger a signaling cascade to effectuate downstream responses. The *in planta* analysis of elements involved in phytohormone signaling can be achieved through transient expression in mesophyll protoplasts, which are a fast and versatile alternative to generating plant lines that stably express a transgene. While promoter-reporter constructs have been used successfully to identify internal or external factors that change phytohormone signaling, the range of available marker constructs does not meet the potential of the protoplast technique for large scale approaches. The aim of our study was to provide novel markers for phytohormone signaling in the Arabidopsis mesophyll protoplast system. We validated 18 *promoter*::*luciferase* constructs towards their phytohormone responsiveness and specificity and suggest an experimental setup for high-throughput analyses. We recommend novel markers for the analysis of auxin, abscisic acid, cytokinin, salicylic acid and jasmonic acid responses that will facilitate future screens for biological elements and environmental stimuli affecting phytohormone signaling.

## Introduction

Elucidating the *in planta* function of genes or regulatory factors is key in the process to understand how individual signaling components are interconnected and contribute to signaling pathways and networks. This task often involves generating transgenic plants which is time-consuming, laborious and cannot easily be applied in large-scale screening approaches. The use of transient gene expression in protoplasts is an alternative technique that offers many advantages such as a high-throughput, cost effectiveness and great flexibility towards the components (e.g. proteins) to be tested [1].

The method is based on the isolation of individual cells from leaf tissue by digesting the surrounding cell walls with the help of fungal enzymes such as cellulase and pectinase. The resulting protoplasts can then be transfected with DNA encoding the proteins of interest through application of osmotic or electric stimuli or by microinjection [2, 3]. While protoplasts are isolated cells and the cellular processes observed may not entirely reflect the complexity of signaling events at the whole plant level, the protoplast system offers versatility and analytic speed which enabled the selection of candidates from larger collections of regulatory elements that would otherwise be difficult to identify [1]. Pioneered by using *Arabidopsis thaliana* leaf mesophyll cells, these advantages have resulted in the establishment of protoplast transient expression assays for multiple species including maize, wheat, tomato, rice and tobacco [4, 5, 6, 7, 8]. Protoplast-based assays have been essential in answering a variety of questions in plant biology and in facilitating the analysis of protein-protein interactions [9], phosphorylation cascades [10], subcellular localization [11] and for testing protein stability [12] or activity [6]. Large-scale approaches using genomic and proteomic methods following cell sorting are now commonly used by the community [13, 14]. In the age of gene editing, protoplasts have been successfully employed in validating the editing efficiency of CRISPR-Cas9 constructs [8]. Several excellent articles offer support for establishing and adapting this method in a new research context [1, 2, 3].

A particularly successful application of the technique are regulation studies between a promoter-reporter construct and an added active agent. Among the elements that have been tested for interaction with promoters in the protoplast system are immunity elicitors [15], transcription factors [1] and microbial effectors [16]. The majority of promoters used in marker constructs for protoplast transient expression assays are known to regulate phytohormone-responsive genes. Plant hormones are essential signaling molecules involved in the coordination of all aspects of plant life including plant growth, development and responses to environmental signals or stresses. Phytohormone perception modulates developmental and metabolic reprogramming in a fast and efficient manner allowing for high plasticity in the responses to different conditions, ranging from nutrient starvation to pathogen attack [17, 18]. Not surprisingly, since most plant processes are tightly controlled by hormonal signaling networks, many studies of plant development or stress integration require the assessment of hormonal responses. In addition to the quantification of phytohormone levels the analysis of downstream changes in gene expression has increased our understanding of hormonal signaling. Phytohormone recognition is mediated by specific receptor proteins residing in different subcellular compartments in the cell. In addition to biochemical fractionation the transient expression of putative receptor proteins in protoplasts has contributed to pinpoint the cellular site of phytohormone perception [19, 20]. Hormone perception by receptors triggers signaling cascades controlling transcriptional regulators which eventually activate or suppress a set of promoters to translate the hormonal stimulus into gene expression changes [21]. The group of Jen Sheen established several phytohormone-responsive markers in the protoplast system. Specifically, the *promoter*::*luciferase* constructs for *RD29A*, *GH3*.*3* and *ARR6* were developed to indicate abscisic acid, auxin and cytokinin signaling in protoplasts, respectively [10, 22]. These reporters have been applied in other studies since then and have provided insights into hormonal signaling following environmental cues such as oxidative stress, high salinity, osmotic stress or immune elicitors [1, 15, 22, 23, 24, 25, 26]. A previous study using the *pRD29A*::*LUC* construct also indicated that *promoter*::*LUC* markers are suited for use in multiwell-based protoplast assays to identify components that change hormonal signaling [1]. However, the range of available phytohormone-responsive promoter constructs is limited and has changed little in the past years, impeding the flexibility of large-scale applications of this technique.

Establishing the protoplast system and new markers for any plant species including Arabidopsis can be challenging and often requires optimization of the isolation and maintenance of the cells as well as identifying the most efficient transfection method. In addition, the specificity of hormone markers is often unclear. The aim of our study was to extend the toolkit of *promoter*::*luciferase* constructs for the analysis of phytohormone responses in the protoplast system and validate the markers under high-throughput conditions. We focused on responses of the phytohormones abscisic acid, auxin, cytokinin, salicylic acid and jasmonic acid due to their principal significance in growth, abiotic stress and disease resistance. We selected 18 promoters based on information in public expression databases and the available literature. In our effort to identify new phytohormone markers, our analyses were guided by two main criteria: validating the responsiveness and specificity of novel as well as previously used promoters in protoplasts. We further present additional criteria that should be considered when developing new marker constructs for a signaling pathway of choice and provide technical details for the establishment of our semi-automated protoplast assay system that is suitable for high-throughput analyses. The presented markers can be co-expressed with a protein of interest, combined with chemical or physical treatments or introduced into protoplasts isolated from a genetic background of choice.

## Materials and methods

### Plant growth

*Arabidopsis thaliana* ecotype Col-0 plants were grown in P24 trays in soil in a controlled environment with 12 h light at 22°C and 12 h dark at 20°C (60% relative humidity). Plants were used for protoplast isolation when 4–5 weeks old.

### Plasmid construction

Hormonal reporter constructs *pRD29A*::*LUC*, *pGH3*.*3*::*LUC*, *pARR6*::*LUC* and *pFRK1*::*LUC* were ordered from ABRC (CD3-912, CD3-913, CD3-917 and CD3-919). The remaining reporter constructs were generated by recombination-based cloning (CloneEZ kit, GenScript). The promoter fragments were amplified by PCR from genomic Col-0 DNA (see primer sequences in S1 Table). The plasmid backbone resulted from digesting *pFRK1*::*LUC* with BamHI and NcoI. The transfection control plasmid was *pAtUBQ10*::*GUS*.

### Protoplast isolation and transfection

The isolation and transfection of mesophyll protoplasts was performed as described previously [1, 2] with the adjustments detailed below. The vacuum infiltration step following transfer of the leaf material into the enzyme solution was omitted. The enzymatic digestion lasted for ~3 hours. Before transfection protoplasts were diluted at 3.3 x 10^5^ cells ml^-1^ in MMG. Protoplast transfection was performed in 96-well plates with a conical bottom (Greiner BioOne 651261). The plasmid containing a specific hormonal reporter (*promoter*::*LUC*) and a transfection control plasmid (*pAtUBQ10*::*GUS*) were added to each well as 1 μl of 1 μg/μl DNA, leaving 2 μg total plasmid DNA in each well. Plasmid DNA was purified using the ZymoPURE plasmid midiprep kit from Zymo Research followed by an additional cleaning step using sodium acetate / ethanol precipitation.

The transfection was performed using the Tecan Freedom EVO200 liquid handling robotic platform but can likewise be carried out manually. The indicated volumes refer to a single well. After adding the plasmid DNA into the wells, 30 μl of protoplasts (~1 x 10^4^) were added before adding 32 μl of PEG 4000 solution. The plate was shaken for 1 min at 1000 rpm and incubated at room temperature for 15 min. After that, 170 μl of W5 solution were added and the plate was shaken for 1 min at 1000 rpm. The plate was then centrifuged at 100 g for 2 min before removing 160 μl of the supernatant. Finally, 140 μl of W1 solution were added before shaking the plate for 1 min at 1000 rpm. Protoplasts were kept in the transfection plates at ambient light conditions (50–100 μmol m^-2^ s^-1^) at 23°C until further analysis the following day. When establishing this assay in a new context, we recommend analyzing the transfection efficiency by transfecting constructs encoding fluorescent proteins and counting the proportion of cells that were transfected. We obtain transfection efficiencies around 50% with the setup described here (S1 Fig).

### Luciferase assay

Expression of the specific hormonal reporter was analyzed by detecting luciferase activity *in vivo*. 100 μl of supernatant were removed from each well. 20 μl of LUC substrate mix were added to each well of a white, round bottom 96-well plate (NUNC U96 PP 267350). Using an 8-channel pipette and cut tips cells were gently resuspended before adding them to the luciferin in the white plate for luminescence reading. Treatments were added using an Eppendorf Multipette^®^ before shaking the plate at 450 rpm. Plates were transferred into the dark 30 min before luminescence reading by a photon-sensitive camera (Photek HRPCS218) for 5.5 hours. Software Image32 (Photek) was used to analyze intensity values. Luminescence derived from the specific hormonal reporter was normalized using GUS activity derived from the transfection control plasmid.

### GUS assay

Expression of the transfection control plasmid was analyzed after the luminescence reading by detecting β-glucuronidase activity in protoplast lysate. Excess supernatant was removed before adding 100 μl of lysis buffer per well and shaking the plate at 450 rpm for 5 min. Plates were centrifuged for 2 min at 1000 g to remove cell debris. 10 μl lysate were transferred to a transparent, flat bottom plate before addition of 100 μl GUS substrate mix. After brief shaking the plate was incubated at 37°C for 1 h before analyzing the fluorescence in a plate reader with excitation at 360 nm and detection at 465 nm.

### Chemicals and reagents

Cellulase R10 and Macerozyme R10 for enzymatic digest of leaf tissue were purchased from Melford Biolaboratories Ltd. Buffer W5 was 154 mM NaCl, 125 mM CaCl_2_, 5 mM KCl, 2 mM MES pH 5.7. MMG solution was 0.4 M mannitol, 15 mM MgCl_2_, 4 mM MES pH 5.7. Buffer W1 was 0.5 M mannitol, 20 mM KCl and 4 mM MES pH 5.7. Substances for hormonal treatments were abscisic acid (ABA), 1-naphthylacetic acid (NAA), trans-zeatin (t-zeatin), salicylic acid (SA) and methyl jasmonate (MeJA) and were purchased from Sigma Aldrich. Mock-treated wells received the amount of solvent present in the medium concentration of the three hormonal treatments or water. For analysis of marker specificity the treatment concentrations were 10 μM ABA, 500 nM NAA, 20 μM t-zeatin, 30 μM SA and 50 μM MeJA.

Lysis buffer was prepared as 5-fold stock solution using 125 mM Tris / H_3_PO_4_ (pH 7.8), 10 mM DTT, 10 mM DACTAA (Sigma D1383), 50% (v/v) glycerol, 5% (v/v) Triton X-100. LUC substrate was prepared using beetle luciferin (Promega E1602) as 1 mM luciferin, 30 mM HEPES (pH 7.8), 3 mM ATP (Sigma 797189) and 15 mM MgSO_4_. GUS substrate was prepared using MUG (4-Methylumbelliferyl-β-D-glucuronide, Melford Biolaboratories Ltd. M65900) as 1 mM MUG, 10 mM Tris / HCl (pH 8.0) and 2 mM MgCl_2_.

## Results and discussion

### Selection of promoters for analysis as hormonal markers in protoplasts

Marker genes are very useful tools in the study and verification of phytohormone pathway regulation and the literature offers many examples of genes known to be induced by phytohormones. Some of these genes are characterized towards their function and position within the signaling network but many have been selected for their consistent transcriptional response to the presence of a phytohormone. Analyzing more than one marker per phytohormonal pathway will ideally provide additional insight into the level at which a regulatory element interferes with the signaling cascade. In this study, we have validated a set of *promoter*::*luciferase* constructs as markers for five different phytohormonal pathways: abscisic acid (ABA), auxin (IAA), cytokinin (CK), salicylic acid (SA) and jasmonic acid (JA). Our goal was to determine the suitability of these markers for their use in high-throughput protoplast transfection assays.

In order to compile a list of potentially suitable phytohormone-responsive genes we searched previously published studies [27–55] and mined available databases for transcriptional information. We were interested not only in the induction levels or responsiveness to the cognate phytohormone but also in their specificity as judged by their response to other phytohormones. Table 1 summarizes information on responsiveness and specificity of 18 genes we considered promising to test for their suitability as markers in the protoplast system.

**Table 1:** Summary of the properties of genes selected as potential markers for phytohormone signaling analyses in protoplasts.

Pathway	Name	ID	Transcriptome repositories		Literature		Induction in protoplasts > 2-fold
			Responsiveness	Specificity	Responsiveness	Specificity	
**ABA**	RD29A	At5g52310	+++	+++	++^1,2^	+++^3^	Yes
	RAB18	At5g66400	+++	+++	+++^4,5^	+++^3^	Yes
**IAA**	GH3.3	At2g23170	+++	+	++^2,6^	+^3^	Yes
	IAA5	At1g15580	+++	+++	+++^7,8^	+++^3,9^	Yes
	IAA29	At4g32280	++	+++	+++^9,10^	+++^3,9^	> 1.5-fold, variable
	LBD29	At3g58190	+	+++	+++^9,11^	+++^3,9^	No
**CK**	ARR6	At5g62920	+	++	+^2,12^	+^3^	Yes
	ARR5	At3g48100	+	++	++^12,13^	++^3,14^	Yes
	NPF2.3	At3g45680	+	+	+^3^	+++^3^	No
	ARR15	At1g74890	+	++	++^12,14^	++^3,14^	No
	CYP735A2	At1g67110	+	++	+^13,15^	++^3,13^	No
**SA**	WRKY70	At3g56400	++	++	++^16^	++^17^	Yes
	LURP1	At2g14560	+++	++	++^18,19^	++^17^	> 1.5-fold, stable
	PR1	At2g14610	++	++	++^17,20^	++^21^	> 1.5-fold, stable
	CBP60G	At5g26920	+	+	+^22^	ND	No
**JA**	JAZ10	At5g13220	++	+++	+++^23,24^	++^3,25^	Yes
	MYB113	At1g66370	+	+++	+++^3,26^	+^3,27^	Yes, but less specific
	PDF1.2	At5g44420	++	+	+^21,28^	+^3,29^	No

More than one gene was selected for each of the five phytohormones. Colors indicate functionality of the respective *promoter*::*luciferase* construct in protoplasts: yellow = functional, grey = requires optimization, white = not suitable. Transcriptional information about the selected genes from Genevestigator and BAR databases (‘transcriptome repositories’) and selected publications (‘literature’) is presented with respect to their specific responsiveness to the cognate phytohormone but no other phytohormones (specificity) [27–55]. Levels of responsiveness and specificity are labelled as low (+), medium (++) or high (+++) correlating with color intensity of the cells. The induction in protoplasts refers to the increase in relative luminescence observed following treatment with the respective hormone.

Using the transcriptome data repositories Genevestigator and BAR (Bio-Analytic Resource for Plant Biology) we analyzed the ability of the studied gene to be specifically induced by the cognate phytohormonal treatment [56, 57]. We also used these databases to gather information on different characteristics of the selected genes, such as their basal expression level in adult leaves and their expression levels observed in leaf or mesophyll cell protoplasts. Candidate genes reported to be strongly induced by protoplasting were avoided. While the expression databases contain large amounts of transcriptional information, the underlying datasets originate from individual studies that were carried out under varying conditions. Therefore, the overview provided in Table 1 represents average experimental settings that may include different plant tissues or developmental stages, as well as varying hormone treatment regimes (e.g. time, concentration). The use of synthetic promoters can be an alternative approach when studying hormonal signaling with protoplasts [58, 59]. Synthetic reporters are likely to be regulated by a reduced set of stimuli when compared to native promoter sequences since they contain fewer regulatory elements. Depending on the experimental question reporters generated with synthetic sequence motifs can be a preferred choice, for example to avoid unwanted crosstalk. However, the extent of their integration in the natural signaling network of the plant cell distinguishes them from reporters based on native promoter sequences. For a limited number of hormonal pathways the use of degradation-based sensors that allow to monitor hormonal perception close to real-time and *in vivo* have also been demonstrated [60, 61]. In order to determine the functionality of the native promoter sequences we generated *promoter*::*LUC* constructs and validated them by protoplast transfection assays.

### Responsiveness of *promoter*::*LUC* reporters to hormone treatment

**Fig 1:**
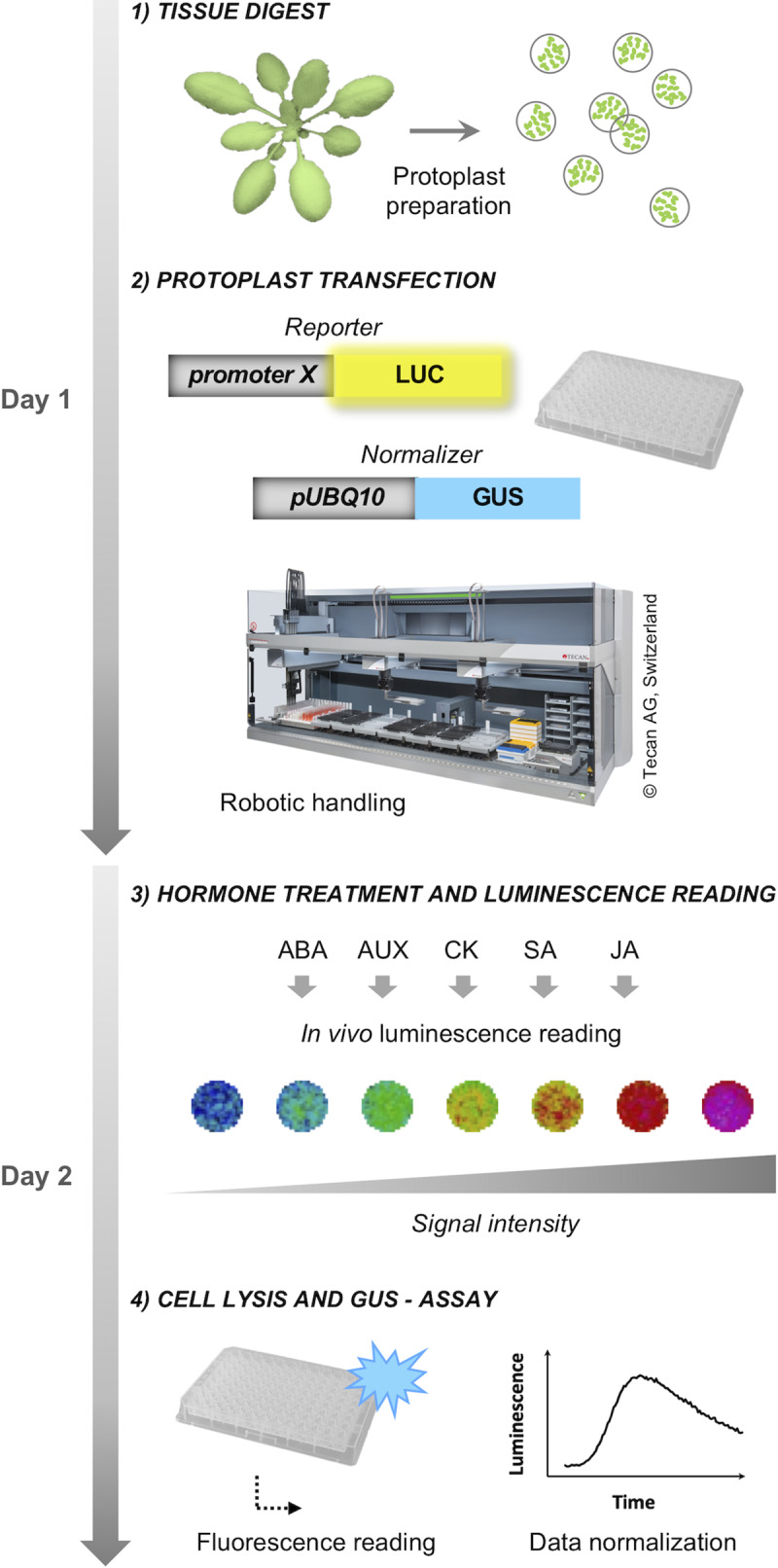
Workflow. Schematic overview of how *promoter*::*luciferase* reporter constructs were tested towards their suitability as phytohormonal markers in protoplasts. Protoplasts were isolated by enzymatic digest of Arabidopsis leaf tissue and transfected with *promoter*::*luciferase* constructs in a 96-well format using a robotic liquid handling platform from Tecan. Activation of the promoters following hormonal treatments was quantified as *in vivo* luminescence (signal) intensity using a photon-sensitive camera. Transfection efficiencies were normalized based on β-glucuronidase activity in cell lysates. LUC, luciferase; GUS, β-glucuronidase.

To determine the responsiveness of hormonal reporters to their cognate phytohormone and the specificity of this response, our experimental workflow monitored the response kinetics of up to 96 samples in parallel (Fig 1).

**Fig 2:**
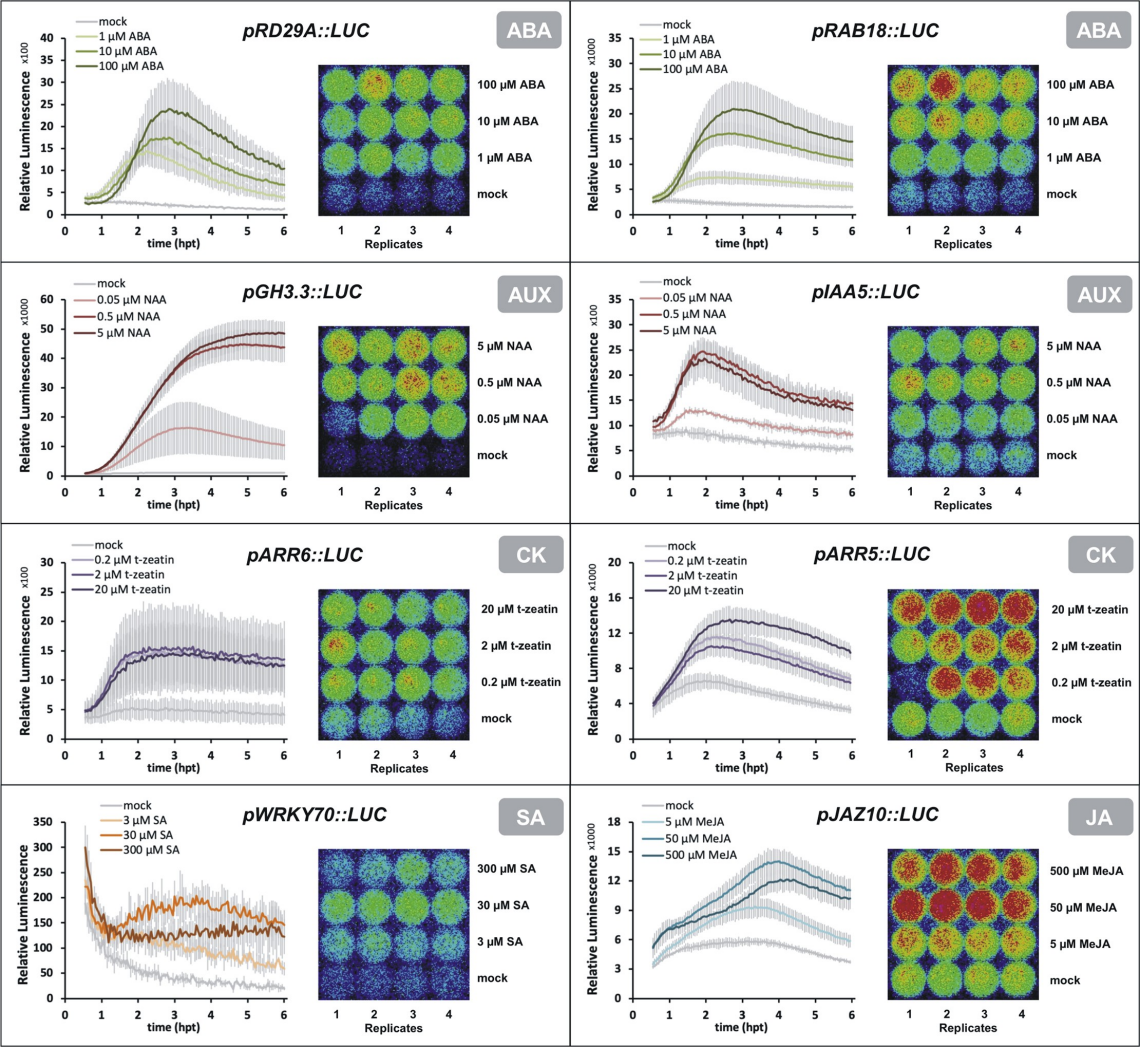
Responsiveness of *promoter*::*luciferase* constructs to phytohormones. Protoplasts were transfected with *promoter*::*luciferase* constructs and treated with the indicated substances to activate phytohormonal signaling using three different concentrations. Luminescence was recorded following phytohormonal treatment for 5.5 hours. The plots on the left of each panel show results from one out of ≥ 3 biological repetitions; error bars represent standard deviations from 3–4 technical replicates. The image on the right of each panel shows the luminescence signal as detected by the photon-sensitive camera. ABA, abscisic acid; AUX, auxin; CK, cytokinin; JA, jasmonic acid; MeJA, Methyl jasmonate; NAA, 1-Naphtaleneacetic acid; SA, salicylic acid; t-zeatin, trans-zeatin; hpt, hours post-treatment.

Protoplasts expressing *promoter*::*LUC* constructs were treated with three concentrations of the cognate phytohormone that differed by a factor of 10 between each other (referred to as ‘low’, ‘medium’ and ‘high’ in the following). Fig 2 summarizes the normalized luminescence monitored from 30 min to 6 hours post-treatment (hpt) and includes a snapshot of the raw luminescence of the replicate samples. All reporter constructs in Fig 2 showed a reproducible fold-change of > 2 between mock and treated samples for at least one of the three tested phytohormone concentrations.

A visible increase in promoter activity was observed around 2 hpt and the intensity of luminescence rarely increased after 5 hpt (Fig 2). As expected, the analyzed markers differed in their responsiveness towards the chosen hormone concentrations. The markers for ABA signaling, *pRD29A*::*LUC* and *pRAB18*::*LUC*, were characterized by high sensitivity where the promoter activity increases with rising treatment concentrations without reaching saturation. In contrast, the response of the two auxin markers *pGH3*.*3*::*LUC* and *pIAA5*::*LUC* did not change significantly between medium and high NAA concentrations. This observation was recently confirmed after treatment with the auxin IAA (indoleacetic acid) using concentrations between 1 and 100 μM IAA in protoplasts transfected with *pGH3*.*3*::*LUC* and *pIAA5*::*LUC* [62]. The responsiveness of the CK markers *pARR6*::*LUC* and *pARR5*::*LUC* did not differ significantly between treatments with 0.2 μM and 2 μM of trans-zeatin across experiments (Fig 2 and S2 Fig), but samples treated with 20 μM trans-zeatin surpassed the 2-fold induction threshold more reliably than the lower concentrations. A marker fold change above 2 makes a screen more sensitive towards weak and medium effects of a tested component, but in experiments where such a fold-change threshold is less critical, the *ARR5* and *ARR6* markers can also successfully be used with low and medium concentrations of trans-zeatin. The *pARR6*::*LUC* construct can be responsive to lower cytokinin treatment concentrations in protoplast assays [63] and the observed difference in responsiveness might possibly be due to aspects of our setup such as the robotic handling. The reporter construct for salicylic acid signaling, *pWRKY70*::*LUC*, had a relatively low promoter activity but showed a stable activation with 30 μM SA. The marker for JA signaling, *pJAZ10*::*LUC*, exhibited variable response kinetics, possibly due to changing effects of wound signaling in the different protoplast preparations. However, *pJAZ10*::*LUC* reproducibly surpassed a 2-fold difference between LUC activity of mock samples and those treated with 50 μM methyl jasmonate (MeJA) at 5 hpt (Fig 2). The integrated marker responses during 5.5 hours were compared as the area under the curve (AUC) and are shown in S2 Fig. Taken together, all markers displayed a responsiveness upon treatment with respective phytohormones. The observed response curves and concentration-dependent differences in responses indicated protoplast integrity and suitability of the assay to quantify phytohormone signaling.

### Specificity of *promoter*::*LUC* reporters during treatment with other hormones

**Fig 3:**
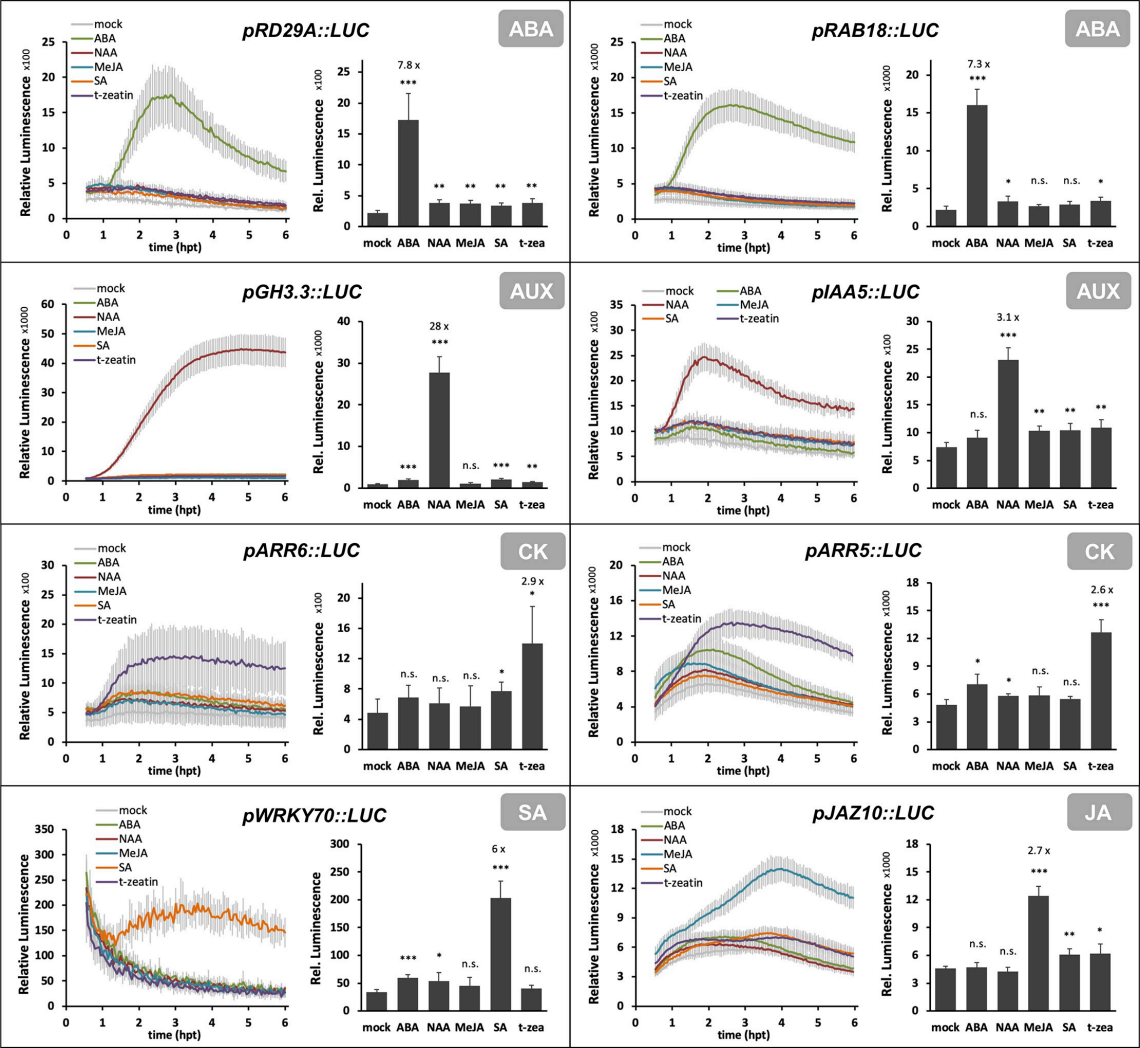
Specificity of *promoter*::*luciferase* constructs in detecting phytohormone signaling. Protoplasts were transfected with *promoter*::*luciferase* constructs and treated with the indicated substances to activate phytohormonal signaling. The following treatments were used: mock, 10 μM ABA, 0.5 μM NAA, 50 μM MeJA, 30 μM SA or 20 μM t-zeatin. Luminescence was recorded following hormonal treatment for 5.5 hours. The plots show results from one out of ≥ 3 biological repetitions; error bars represent standard deviations from 3–4 technical replicates. Bar charts show the relative luminescence of the *promoter*::*luciferase* constructs at the following timepoints (hours post treatment, hpt): *pRD29A*::*LUC* (2.5hpt), *pRAB18*::*LUC* (2.5hpt), *pGH3*.*3*::*LUC* (2.5hpt), *pIAA5*::*LUC* (2.5hpt), *pARR6*::*LUC* (4hpt), *pARR5*::*LUC* (4hpt), *pWRKY70*::*LUC* (3hpt), *pJAZ10*::*LUC* (5hpt). Statistical analysis was performed using Student’s t-test: * p < 0.05, ** p < 0.01, *** p < 0.001. ABA, abscisic acid; AUX, auxin; CK, cytokinin; JA, jasmonic acid; MeJA, Methyl jasmonate; NAA, 1-Naphtaleneacetic acid; SA, salicylic acid; t-zeatin, trans-zeatin.

Although marker specificity is essential, such information is rarely provided for protoplast assays. The specificities of the marker gene responses were analyzed by testing each reporter against the five phytohormones used in this study. All 8 *promoter*::*LUC* reporters showed a specific induction with the cognate phytohormone when compared to the other 4 tested substances. Fig 3 shows the development of normalized luminescence over time and analyzes the samples at the timepoint when the marker response exhibits a strong fold-change between mock samples and cells treated with the cognate phytohormone while maintaining high specificity towards the other phytohormones. These timepoints are recommended for experimental setups which do not require continuous monitoring of the marker responses after treatment: 2.5 hours post treatment (hpt) for the ABA markers *pRD29A*::*LUC* and *pRAB18*::*LUC* and the auxin markers *pGH3*.*3*::*LUC* and *pIAA5*::*LUC*, 3 hpt for the SA marker *pWRKY70*::*LUC*, 4 hpt for the CK markers *pARR6*::*LUC* and *pARR5*::*LUC* and 5 hpt for the JA marker *pJAZ10*::*LUC*. It is important to emphasize that the analytic conditions defined here provide reliable guidelines for using these *promoter*::*LUC* reporters, though individual experimental setups will benefit from further optimization in the given laboratory and experimental environment.

The results presented in Figs 2 and 3 also demonstrated that it cannot generally be predicted from basal gene expression levels whether or not a promoter is suited for the use in reporter constructs. An example are the SA-markers *pWRKY70*::*LUC* and *pPR1*::*LUC*, where the latter shows a high basal activity but a low responsiveness to SA whereas *pWRKY70*::*LUC* produces a strong and reproducible induction after SA treatment although the promoter-derived luminescence will remain low (Fig 2 and S3 Fig). Although the present study validated the specificity of 8 markers towards five different phytohormones, some of the markers might be induced by other hormonally active substances or additional stimuli such as environmental or metabolic clues which could not be covered here. We have clearly shown that the presented markers are specifically induced by the phytohormone they were selected for and not by any of the other hormones used in this study.

### Additional *promoter*::*LUC* constructs with decreased responsiveness

Among the 15 newly generated *promoter*::*luciferase* constructs tested for their suitability as hormonal markers in protoplast-based assays we evaluated 3 as requiring further optimization and 7 as not suitable based on their responsiveness to different concentrations of the cognate hormone (S3 Fig). Some reporters, such as *pIAA29*::*LUC* (auxin), *pLURP1*::*LUC* (SA) and *pPR1*::*LUC* (SA) were activated following respective phytohormone treatments but did not reproducibly surpass the 2-fold threshold we set as recommended standard for our analyses. The JA marker *pMYB113*::*LUC* responded with a fold-change of 2–3 in 3 of 4 experiments and can be recommended for use in those cases where *pJAZ10*::*LUC* is not a preferred choice.

The three CK markers *pNPF2*.*3*::*LUC*, *pARR15*::*LUC* and *pCYP735A2*::*LUC* seemed generally unresponsive. The markers used in this study might respond differently when isolated by other protoplasting methods [3] and can also be analyzed towards changes in their basal activity in the presence of additional factors such as chemicals, transcriptional regulators and many others.

## Conclusions

The *in planta* analysis of phytohormone signaling is a time-consuming and often low-throughput process. We generated new phytohormone markers for the Arabidopsis leaf protoplast system and validated 5 novel and 3 previously used markers as suitable for quantitative analyses of hormonal responses *in planta*. These markers can be used to analyze hormonal signaling after treatments with chemicals, environmental stimuli or endogenous and exogenous effectors and also allow to compare signaling in different mutant genotypes. When working with a 96-well format we comfortably processed 4 plates per experiment, resulting in a throughput of around 170 active agents tested against a hormone-responsive promoter of choice in duplicate samples. The unique feature of the protoplast transfection system is its flexibility towards the pathway of interest determined by the experimental reporter, making it an excellent method for biological screens. It can be anticipated that additional, bespoke markers for other areas of plant research will expand the applications of the protoplast expression system in the future.

## Supporting information

S1 Fig Transfection efficiency of protoplasts using 96-well plates and robotic handling. Bars indicate the percentage of protoplasts transfected with a *35S*::*mCherry* construct in the pool of total protoplasts as determined by counting of ≥ 100 cells in each independent transfection sample. The experiment was repeated three times (exp1-3) with 8 independent transfection samples for each experiment. Error bars represent the standard error (n = 8).
(TIFF)

S2 Fig Responsiveness of *promoter::luciferase* constructs as area under the curve (AUC). Integration of the signals from experiments analyzing responsiveness of the markers shown in Fig 2 over 5.5 hours. The plots show results from one out of ≥ 3 biological repetitions; error bars represent standard deviations from 3–4 technical replicates. Statistical analysis was performed using Student’s t-test: * p < 0.05, ** p < 0.01, *** p < 0.001. ABA, abscisic acid; AUX, auxin; CK, cytokinin; JA, jasmonic acid; MeJA, Methyl jasmonate; NAA, 1-Naphtaleneacetic acid; SA, salicylic acid; t-zeatin, trans-zeatin.
(TIFF)

S3 Fig Additional markers tested. Protoplasts were transfected with *promoter*::*luciferase* constructs and treated with the indicated substances to activate hormonal signaling using three different concentrations. Luminescence was recorded following hormonal treatment for 5.5 hours. The plots show results from one out of ≥ 2 biological repetitions; error bars represent standard deviations from 3–4 technical replicates. AUX, auxin; CK, cytokinin; JA, jasmonic acid; MeJA, Methyl jasmonate; NAA, 1-Naphtaleneacetic acid; SA, salicylic acid; t-zeatin, trans-zeatin; hpt, hours post-treatment.
(TIFF)

S1 Table Primer sequences used for PCR-amplification of the promoter fragments. (PDF)
