# Supplementary figures and images for: Novel markers for high-throughput protoplast-based analyses of phytohormone signaling
Source: PLoS One. 2020 Jun 4;15(6):e0234154. doi: 10.1371/journal.pone.0234154 (PMC7272087; doi:10.1371/journal.pone.0234154)

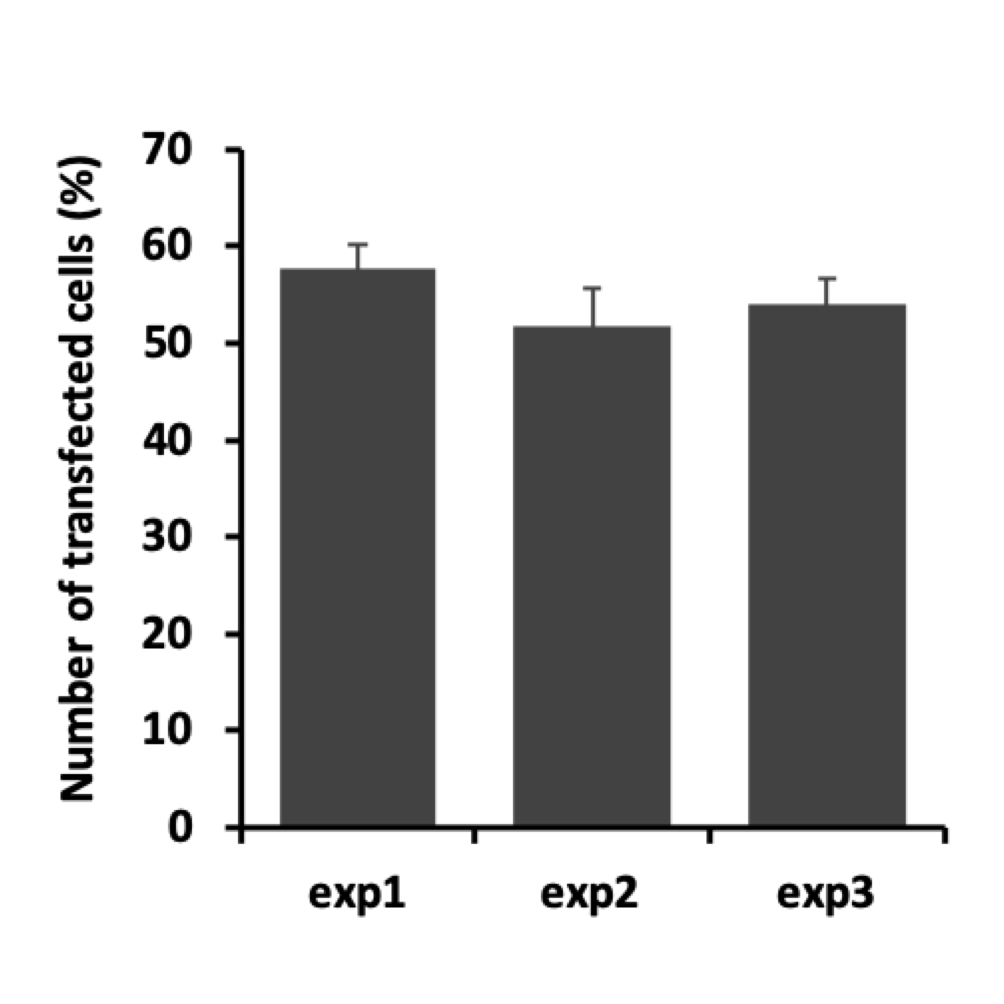

Supplement: S1 Fig — Bars indicate the percentage of protoplasts transfected with a 35S::mCherry construct in the pool of total protoplasts as determined by counting of ≥ 100 cells in each independent transfection sample. The experiment was repeated three times (exp1-3) with 8 independent transfection samples for each experiment. Error bars represent the standard error (n = 8). (TIFF) [file pone.0234154.s001.tiff]

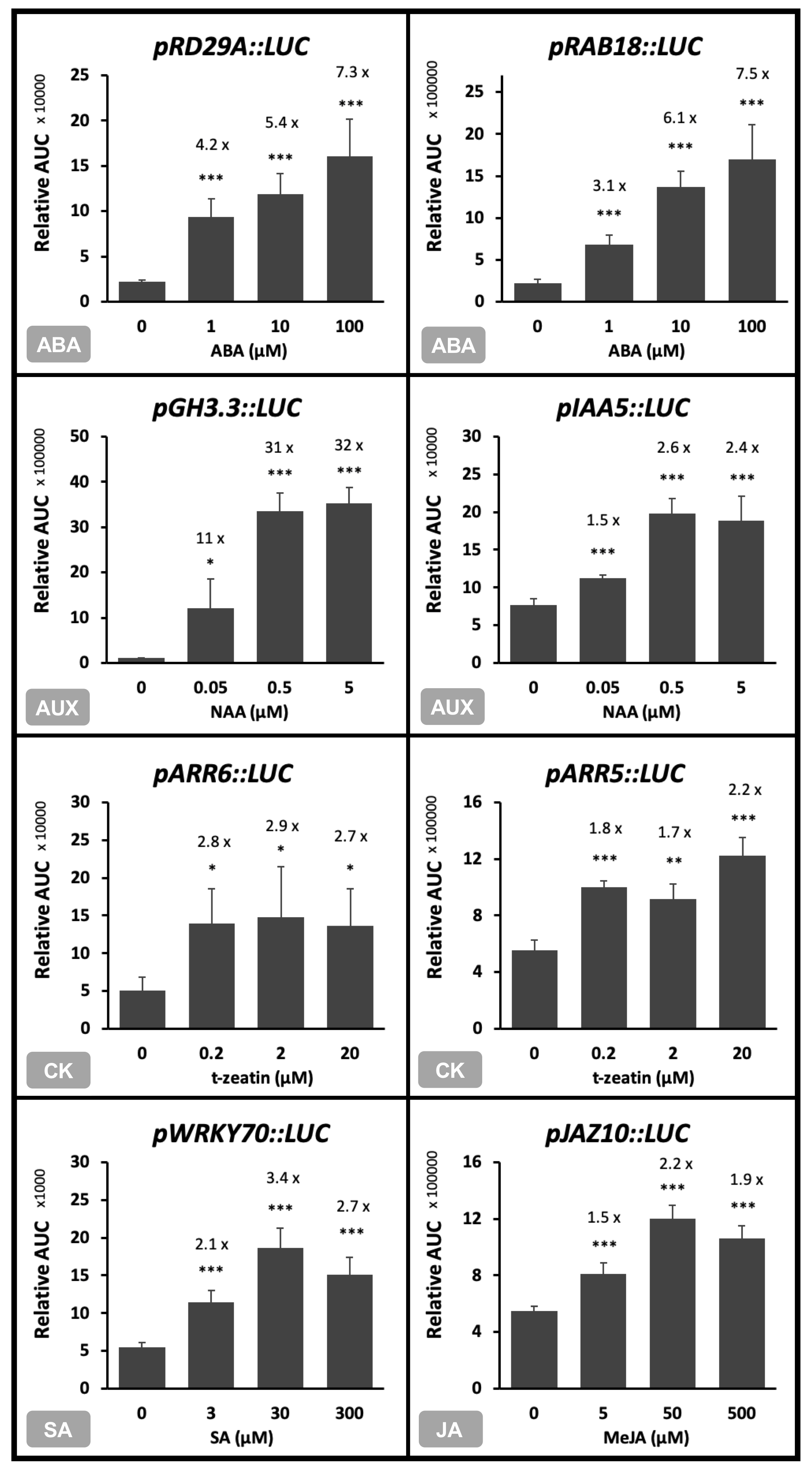

Supplement: S2 Fig — Integration of the signals from experiments analyzing responsiveness of the markers shown in Fig 2 over 5.5 hours. The plots show results from one out of ≥ 3 biological repetitions; error bars represent standard deviations from 3–4 technical replicates. Statistical analysis was performed using Student’s t-test: * p < 0.05, ** p < 0.01, *** p < 0.001. ABA, abscisic acid; AUX, auxin; CK, cytokinin; JA, jasmonic acid; MeJA, Methyl jasmonate; NAA, 1-Naphtaleneacetic acid; SA, salicylic acid; t-zeatin, trans-zeatin. (TIFF) [file pone.0234154.s002.tiff]

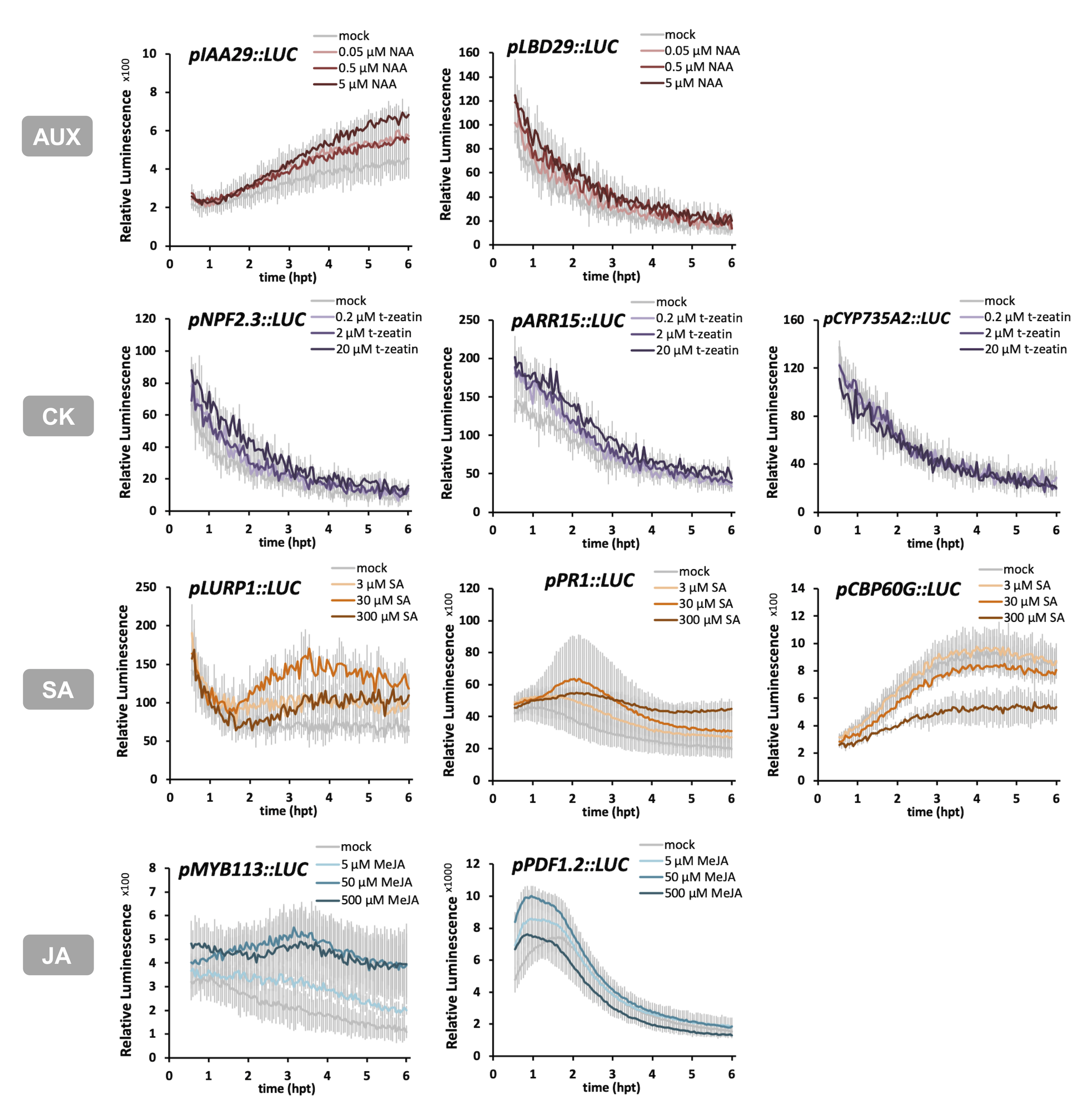

Supplement: S3 Fig — Protoplasts were transfected with promoter::luciferase constructs and treated with the indicated substances to activate hormonal signaling using three different concentrations. Luminescence was recorded following hormonal treatment for 5.5 hours. The plots show results from one out of ≥ 2 biological repetitions; error bars represent standard deviations from 3–4 technical replicates. AUX, auxin; CK, cytokinin; JA, jasmonic acid; MeJA, Methyl jasmonate; NAA, 1-Naphtaleneacetic acid; SA, salicylic acid; t-zeatin, trans-zeatin; hpt, hours post-treatment. (TIFF) [file pone.0234154.s003.tiff]
