# Supplementary material for: Novel markers for high-throughput protoplast-based analyses of phytohormone signaling
Source: PLoS One. 2020 Jun 4;15(6):e0234154. doi: 10.1371/journal.pone.0234154 (PMC7272087; doi:10.1371/journal.pone.0234154)
Supplement: S1 Table — (PDF) [file pone.0234154.s004.pdf]

S1 Table: Primer sequences used for PCR-amplification of the promoter fragments.

Promoter	Gene ID	Size	Primers 5' > 3'
RAB18	At5g66400	650 bp	CCTGCAGGCTCTAGAGGATCCGGTGCAGAGAGGAGAAACTCCC<br>TGTTTTTGGCGTCTTCCATGGTTCTTGTCTTAAGCAAACACTTTGAG
IAA5	At1g15580	920 bp	CCTGCAGGCTCTAGAGGATCCGCTGTCCATTATCACAAAGTC<br>TGTTTTTGGCGTCTTCCATGGCTTTGATGTTTTTGATTGAAAG
ARR5	At3g48100	1650 bp	CCTGCAGGCTCTAGAGGATCCCCAATAAAGCATATTTGTCTTCAGC<br>TGTTTTTGGCGTCTTCCATGGATCAAGAAGAGTAGGATCGTGACTC
WRKY70	At3g56400	2060 bp	CCTGCAGGCTCTAGAGGATCCCAACTGGTTTATCATAGTATC<br>TGTTTTTGGCGTCTTCCATGGGTATCCATTTGTTAGTTTTGAG
JAZ10	At5g13220	2010 bp	CCTGCAGGCTCTAGAGGATCCCAACTATAGGTTCAATTATATTC<br>TGTTTTTGGCGTCTTCCATGGCTTCTTTGATCTTATTAGAAAG
IAA29	At4g32280	2460 bp	CCTGCAGGCTCTAGAGGATCCGTAAGTTTTGCTAGTAGTCATG<br>TGTTTTTGGCGTCTTCCATGGTTCTAAGGCAGCTTCGTCTTTGA
LBD29	At3g58190	2230 bp	CCTGCAGGCTCTAGAGGATCCATGACGATACGAATTCTAAG<br>TGTTTTTGGCGTCTTCCATGGCATCCCTTTGCACATTTCCTC
NPF2.3	At3g45680	1480 bp	CCTGCAGGCTCTAGAGGATCCGTTATATATGTTTGGTGCAAC<br>TGTTTTTGGCGTCTTCCATGGGATTTGAGCTTCTTTATCAC
ARR15	At1g74890	1220 bp	CCTGCAGGCTCTAGAGGATCCCGATTGGTTTGTTTTATTTTG<br>TGTTTTTGGCGTCTTCCATGGCTCTCAATAACTTTACGATC
CYP735A2	At1g67110	2040 bp	CCTGCAGGCTCTAGAGGATCCGTCACATTACAGTTTGTTTAG<br>TGTTTTTGGCGTCTTCCATGGGACTCTTAATATCAAGGTCATG
LURP1	At2g14560	1190 bp	CCTGCAGGCTCTAGAGGATCCAAGTTAGTTATAAAATACTG<br>TGTTTTTGGCGTCTTCCATGGCTGCTGCATACTTTGTTTTC
PR1	At2g14610	1320 bp	CCTGCAGGCTCTAGAGGATCCCCTATGGTGTCATTTTATAAGTTAGCAC<br>TGTTTTTGGCGTCTTCCATGGTTTTCTAAGTTGATAATGGTTATTGTTG
CBP60G	At5g26920	1240 bp	CCTGCAGGCTCTAGAGGATCCGTTACTTTAGTTTTCTGAATATC<br>TGTTTTTGGCGTCTTCCATGGTGATCACTTTTAGGTTTAGAG
MYB113	At1g66370	1030 bp	CCTGCAGGCTCTAGAGGATCCATCGACTCTATACTACAAGA<br>TGTTTTTGGCGTCTTCCATGGTCCATGTACCTTTTCTCAAC
PDF1.2	At5g44420	550 bp	CCTGCAGGCTCTAGAGGATCCCGCGGAACAATGCTGCTCTTG<br>TGTTTTTGGCGTCTTCCATGGGATGATTATTACTATTTTGTTTTCAATGTA
